# Relaxing the assumption of constant transition rates in a multi-state model in hospital epidemiology

**DOI:** 10.1186/s12874-020-01192-8

**Published:** 2021-01-11

**Authors:** Micki Hill, Paul C. Lambert, Michael J. Crowther

**Affiliations:** 1 Biostatistics Research Group, Department of Health Sciences, University of Leicester, George Davies Centre, University Road, Leicester, LE1 7RH UK; 2 Department of Medical Epidemiology and Biostatistics, Karolinska Institutet, Box 281, Stockholm, 17177 Sweden

**Keywords:** Multi-state models, Competing risks, Survival analysis, Markov processes, Transition probabilities, Expected length of stay, Software

## Abstract

**Background:**

Multi-state models are being increasingly used to capture complex disease pathways. The convenient formula of the exponential multi-state model can facilitate a quick and accessible understanding of the data. However, assuming time constant transition rates is not always plausible. On the other hand, obtaining predictions from a fitted model with time-dependent transitions can be challenging. One proposed solution is to utilise a general simulation algorithm to calculate predictions from a fitted multi-state model.

**Methods:**

Predictions obtained from an exponential multi-state model were compared to those obtained from two different parametric models and to non-parametric Aalen-Johansen estimates. The first comparative approach fitted a multi-state model with transition-specific distributions, chosen separately based on the Akaike Information Criterion. The second approach was a Royston-Parmar multi-state model with 4 degrees of freedom, which was chosen as a reference model flexible enough to capture complex hazard shapes. All quantities were obtained analytically for the exponential and Aalen-Johansen approaches. The transition rates for the two comparative approaches were also obtained analytically, while all other quantities were obtained from the fitted models via a general simulation algorithm. Metrics investigated were: transition probabilities, attributable mortality (AM), population attributable fraction (PAF) and expected length of stay. This work was performed on previously analysed hospital acquired infection (HAI) data. By definition, a HAI takes three days to develop and therefore selected metrics were also predicted from time 3 (delayed entry).

**Results:**

Despite clear deviations from the constant transition rates assumption, the empirical estimates of the transition probabilities were approximated reasonably well by the exponential model. However, functions of the transition probabilities, e.g. AM and PAF, were not well approximated and the comparative models offered considerable improvements for these metrics. They also provided consistent predictions with the empirical estimates in the case of delayed entry time, unlike the exponential model.

**Conclusion:**

We conclude that methods and software are readily available for obtaining predictions from multi-state models that do not assume constant transition rates. The multistate package in Stata facilitates a range of predictions with confidence intervals, which can provide a more comprehensive understanding of the data. User-friendly code is provided.

**Supplementary Information:**

The online version contains supplementary material available at (10.1186/s12874-020-01192-8).

## Background

Multi-state models are being increasingly used to investigate complex disease pathways, for example, when interest lies in subsequent and/or intermediate events as well as a primary event. This unified approach facilitates a better understanding of the whole disease profile and provides clinically relevant predictions, for example, transition probabilities and expected duration in each state. One example is in breast cancer, where the time to intermediate events, such as local recurrence and distant metastases, is of interest as well as overall survival [1]. Another example is repeated hospitalisations in patients with heart failure, where interest lies in the time spent in hospital (during each episode and in total) [2]. Further applications include other cancers (colorectal [3, 4], ovarian [5] and acute myeloid leukemia [6]), progression to diabetes [7], health-care associated urinary tract infections [8] and pleural effusion following allogeneic hematopoietic stem cell transplantation [9].

Semi- and non-parametric methods have been frequently used to analyse multi-state models, however, interest is growing in parametric approaches. Although a variety of complex parametric models can relatively easily be fitted to each transition; the difficulty lies in obtaining the corresponding predictions from the full multi-state model. Assuming an exponential Markov model allows for direct calculation of the transition probabilities, as the Kolmogorov forward equations can be solved analytically, however, assuming constant transition rates can be restrictive. Piecewise exponential models relax this assumption [10], however, discontinuous transition rates may not be biologically plausible. Another suggestion has been to model the transition rates of a Markov model with quadratic B-splines and obtain predictions by numerically solving the Kolmogorov forward equations [11]. This paper focuses on a general simulation algorithm to obtain predictions from a range of fitted parametric models [12], including Royston-Parmar models [13]. In terms of implementation, available software includes: mstate in R [14] for semi- and non-parametric methods; msm in R [10] for exponential and piecewise exponential models; flexsurv in R [15] for fitting models and obtaining predictions by numerically solving the Kolmogorov forward equations; and flexsurv in R [15] or multistate in Stata [12] for the general simulation algorithm, the latter following model fitting by merlin [16, 17].

Von Cube et al. [18] recommended the exponential model as an accessible approach to obtain a quick, general understanding of the data. The authors demonstrated this method on hospital acquired infection (HAI) data: an extended illness-death model where a patient can have a HAI (intermediate event) and then/or be discharged or die (competing risks, the death/discharge with HAI are distinct from those without, resulting in 6 states). Von Cube et al. [18] acknowledged the potential implausibility of time constant transition rates (saying that this assumption is rarely met in practice) and recommended more sophisticated methods if the assumption was violated.

In this paper, we compare the method described in von Cube et al. [18] to the approach described in Crowther and Lambert [12]. We demonstrate that there is accessible software - namely the multistate package in Stata [12] - to obtain transition probabilities from multi-state models with transition-specific (and time-dependent) distributions. We then extend the previous analysis [18] by presenting, and highlighting the importance of, uncertainties and by estimating length of stay.

This paper is organised as follows: the “Methods” section introduces the multi-state process, illustrative example, metrics of interest and analysis approaches. The “Results” section displays the quantities of interest graphically, including comparisons between the approaches and confidence intervals for the predictions. The “Discussion” section provides recommendations for future analyses. User-friendly code for the illustrative example is provided in Additional file 1.

## Methods

### Multi-state models

Following Fiocco et al. [19], consider a stochastic process *Y*(*t*),*t*≥0 with a finite state space $ \mathcal {Z} = \{1,\ldots,Z\} $ and process history up to time $ s, \mathcal {H}_{s} = \{ Y(u); 0 \leq u \leq s \} $. The transition probabilities can then be defined as:

1 $$  P(Y(t)=b|Y(s)=a,\mathcal{H}_{s-})  $$

This is the probability that a patient in state *a* at time *s* moves to state *b* by time *t*, conditional on the process history up until the time just before $ s, \mathcal {H}_{s-} $, where $ a, b \in \mathcal {Z} $. This can be simplified to a Markov model, which makes the assumption that the probability in Eq. 1 is only conditional on the state at time *s* and no other process history:

2 $$  {}P\left(Y(t) = b | Y(s) = a, \mathcal{H}_{s-}\right) = P(Y(t) = b | Y(s) = a)  $$

Henceforth, let *P*_*ab*_(*s*,*t*) represent the transition probability given in Eq. 2. This paper focuses on Markov models.

The transition rate, or transition hazard, from state *a* to state *b* at time *t* is:

$$h_{ab}(t) = \underset{\delta t\to 0}{\lim} \frac{P(Y(t+\delta t) = b|Y(t) = a)}{\delta t} $$

This represents the instantaneous failure rate of moving from state *a* to *b* and is analogous to the hazard function in the standard survival setting. The collection of transition rates governs the rate at which patients move between states and therefore the multi-state model. For a tutorial in multi-state models see Putter et al. [20].

Another useful measure is the restricted length of stay in a state. This is analogous to restricted mean survival in the standard survival setting [21]. The residual, restricted expected length of stay in state *b* given a patient is in (non-absorbing) state *a* at time *s* is:

$$e_{ab} (s,t) = \int_{s}^{t} P(Y(u) = b | Y(s) = a) du $$

See Grand and Putter [22] for more details on expected length of stay.

### The extended illness-death model for HAIs

**Fig. 1:**
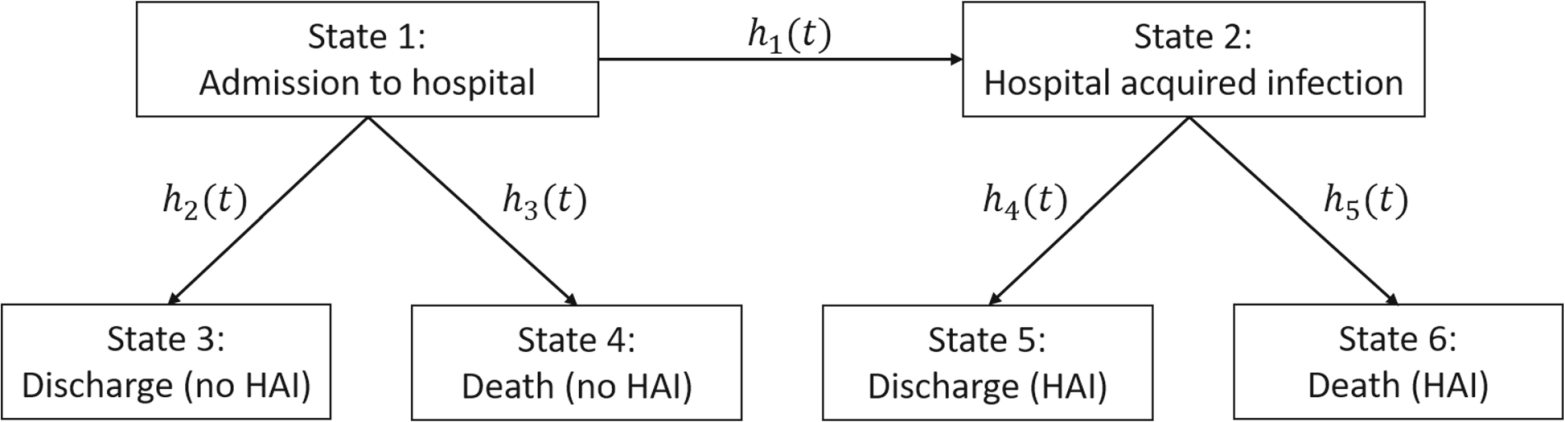
Extended illness-death model for discharge and death with and without a hospital acquired infection (HAI)

This paper considers a multi-state model in the context of hospital acquired infections (HAIs), as previously described by von Cube et al. [18]. When a patient is admitted to hospital, they are at risk of acquiring a HAI, which could lead to an increased hospital stay or increased risk of (hospital) death. An extended illness-death model with six states and five transitions, as illustrated in Fig. 1, has been used to investigate the risks and consequences of HAIs. The time scale is days since hospital admission. All patients begin in state 1 at time 0, where the patient has been admitted to hospital but does not have an infection. The patient will then either become infected (state 2), be discharged without an infection (state 3) or die without an infection (state 4). If the patient acquires an infection, they will then either be discharged (state 5) or die (state 6) with an infection. The *i*^*th*^ transition rate from state *a*_*i*_ to *b*_*i*_ has been denoted as *h*_*i*_(*t*) where:

$$\begin{array}{*{20}l} \left\{a_{1},a_{2},a_{3},a_{4},a_{5}\right\} = \{1,1,1,2,2\} \\ \{b_{1},b_{2},b_{3},b_{4},b_{5}\} = \{2,3,4,5,6\} \end{array} $$

### Metrics of interest

The first metric of interest was transition probabilities from state 1 at time 0, *P*_1*b*_(0,*t*),*b*={1,2,3,4,5,6}. By definition, HAIs take at least three days to develop [18] and so there were no HAI events prior to time 3 (3 days after hospital admission). Therefore, transition probabilities from state 2 at time 3, *P*_2*b*_(3,*t*),*b*={2,5,6}, were also estimated.

Following the formulas from Schumacher et al. [23] and von Cube et al. [18], let *P*_1*D*_(*s*,*t*) denote the probability of dying (states 4 or 6) by time *t*, given a patient was in hospital without a HAI (state 1) at time *s*. Let *P*_16|+_(*s*,*t*) denote the probability of dying with a HAI (state 6) by time *t*, given a patient was in hospital without a HAI (state 1) at time *s* and had become infected (states 2, 5 or 6) by time *t*. Similarly, let *P*_14|−_(*s*,*t*) denote the probability of dying without a HAI (state 4) by time *t*, given a patient was in hospital without a HAI at time *s* and in a non-infected state (states 1, 3 or 4) at time *t*. The quantities can be calculated as follows:

$$\begin{array}{*{20}l} P_{1D}(s,t) & = P(Y(t)\in \{4,6\} | Y(s) = 1) \\ & = P_{14}(s,t) + P_{16}(s,t) \end{array} $$

$$\begin{array}{*{20}l} {}P_{16|+}(s,t) & = P(Y(t)=6|Y(s)=1, Y(t) \in \{2,5,6\}) \\ & = \frac{P_{16}(s,t)}{P_{12}(s,t) + P_{15}(s,t) + P_{16}(s,t)} \end{array} $$

$$\begin{array}{*{20}l} {\kern-6.2pt}P_{14|-}(s,t) & = P(Y(t)=4|Y(s)=1, Y(t) \in \{1,3,4\}) \\ & = \frac{P_{14}(s,t)}{P_{11}(s,t) + P_{13}(s,t) + P_{14}(s,t)} \end{array} $$

The second set of metrics of interest were attributable mortality (AM) and population attributable fraction (PAF). AM and PAF can be used to investigate the excessive risk of dying due to HAIs, see references for discussion [18, 23].

3 $$  AM(s,t) = P_{16|+}(s,t) - P_{14|-}(s,t)  $$

4 $$  PAF(s,t) = \frac{P_{1D}(s,t) - P_{14|-}(s,t)} { P_{1D}(s,t) }  $$

AM and PAF were estimated from time 0 (i.e. *s*=0 in Eqs. 3 and 4).

The third metric of interest was expected length of stay. Given a patient started in state 1 at time 0, the following quantities were estimated: restricted length of stay in state 1 (*e*_11_(0,*t*)), in state 2 (*e*_12_(0,*t*)) and overall in hospital (*e*_11_(0,*t*)+*e*_12_(0,*t*)). Due to the three day delay in developing a HAI, the residual, restricted length of stay in state 2, conditional on having entered state 2 by time 3, was also of interest (*e*_22_(3,*t*)). These were calculated up until the last event time (from any transition).

### Analysis approaches

The three (set of) metrics were obtained from three models. The predictions from the different models were compared against each other and against non-parametric Aalen-Johansen estimates. Note that we use the terms “Aalen-Johansen” and “empirical” interchangeably to refer to the non-parametric estimates. The Aalen-Johansen estimator generalises the Kaplan-Meier estimator to Markov multistate processes. The metrics are obtained analytically, see Additional file 2 for brief details or the following references for more comprehensive details [24–26].

The first approach was to fit an exponential model to each of the transitions and obtain the metrics analytically, as was demonstrated by von Cube et al. [18]. This approach was referred to as the “Exp” model. The parametrisation of all three models is presented in Additional file 2.

The second approach was to select the best fitting distribution for each transition based on the Akaike Information Criterion (AIC), henceforth denoted the “AIC” model. Following Crowther and Lambert [12], to each transition the following parametric models were applied: exponential, Weibull, Gompertz, log-logistic, log-normal, generalised gamma and Royston-Parmar models [13] with 2 to 5 degrees of freedom. The confidence intervals for the transition rates were obtained using the delta method. Once the multi-state model was fitted analytically, with the best fitting distribution for each transition, the general simulation algorithm was applied to obtain the metrics, see the next section for more details.

The third approach was to fit a Royston-Parmar model with 4 degrees of freedom to each of the transitions, henceforth denoted the “RP(4)” model. This was chosen as a reference parametric model for comparison purposes, as it should have sufficient flexibility to capture most complex hazard shapes. A recent sensitivity analysis of Royston-Parmar models [27] suggested that 4 degrees of freedom can adequately capture the baseline hazard. The analysis was performed in a relative survival setting; however, the conclusions can be applied to standard survival [27]. Once the multi-state model was fitted analytically, the general simulation algorithm was applied to obtain the metrics.

### Simulation algorithm

The simulation algorithm works by projecting a patient through the multi-state model in order to create their full event history. This is done a large number of times and the metrics of interest are calculated empirically from the large complete set of histories. The process will now be described in brief, for further details see Crowther and Lambert [12]. Let *a* be the starting state, entered at time *t*_*a*_. If desired, specify a maximum follow-up time *t*_*max*_. For each simulated patient, following the algorithm of Fiocco et al. [19] and Crowther and Lambert [12], repeat the following:

1. Let $ \mathcal {B} $ be the set of states that can be reached from state *a* and let *N*_*a*_ be the cardinality of set $ \mathcal {B} $. If *N*_*a*_=0 (i.e. *a* is an absorbing state), stop. Otherwise, for each state $ b \in \mathcal {B} $, let *h*_*ab*_(*t*) represent the transition rate from *a*→*b*.
2. For each state $ b \in \mathcal {B} $, use *h*_*ab*_(*t*) to simulate event times $ t^{*}_{ab} $ conditional on entering state *a* at time *t*_*a*_. Event times are simulated using the general inversion method described in Crowther and Lambert [28].
3. The observed event time is then $ t^{*} = min\left \{t^{*}_{a1},\ldots,t^{*}_{aN_{a}},t_{max}\right \} $. If *t*^∗^=*t*_*max*_, stop.
4. Set *a*=*c* where $ t^{*} = t^{*}_{ac}, c \in \mathcal {B} $ and set *t*_*a*_=*t*^∗^.

The algorithm above is repeated for a large *N* number of patients. The transition probabilities are then estimated by calculating the proportion of simulated patients in each state at each time point of interest. The full event history of the simulated patients is known and therefore extended predictions can easily be obtained. For example, expected length of stay can be calculated by averaging the time spent in each state (up to each time point of interest) over all patients.

Let $ \hat {\mathbf {b}} $ be the vector of parameter estimates and $ \hat {\mathbf {V}} $ be the variance-covariance matrix from the fitted multi-state model (note that it is $ \hat {\mathbf {b}} $ that is used to obtain the transition rates *h*_*ab*_(*t*) in the algorithm above). Confidence intervals can be obtained by drawing from a multivariate normal distribution with mean $ \hat {\mathbf {b}} $ and variance $ \hat {\mathbf {V}} M $ times [12, 19]. For each draw *m*, the simulation algorithm above is repeated using the sampled ${\hat {\mathbf {b}}_{m}}$ instead of $ \hat {\mathbf {b}} $ to calculate the transition rates (and therefore event times). The variance of the *M* sets of estimates is then calculated and used to produce confidence intervals via normal approximation.

When the general simulation approach was utilised, 1000000 simulated patients were used (*N*=1000000) for the point estimates. 100000 simulated patients (*N*=100000) were repeated 500 times (*M*=500) for the corresponding confidence intervals. These values were chosen as a balance to minimise the Monte Carlo error and computational time. To produce the confidence intervals for the transition probabilities from the “AIC” model, where probabilities were calculated at 165 equally spaced time points, took 48.5 minutes on a standard HP laptop with i5 processor and 8 GB of RAM.

### Software

All analyses were performed in Stata version 15.1 and the code can be found in Additional file 1. The parametric transition rates were obtained using merlin, version 1.12.0 dated 20/09/2020. All predictions and confidence intervals obtained via the general simulation algorithm were achieved using predictms, version 4.0.0 dated 28/10/2020, from the multistate package. The Aalen-Johansen estimates were obtained using msaj, version 1.0.1 dated 11/09/2020, also part of the multistate package.

## Results

### Data

The analysis was performed on the publicly available los.data from the R package etm [24]. This is a sample from an observational cohort study conducted to analyse the burden of HAIs in intensive care, see Beyersmann et al. [29] for details. 756 patients were admitted to hospital (all patients started in state 1 at time 0). 632 patients did not acquire an infection during the study, of which 475 were discharged and 157 died. 124 patients did acquire an infection, of which 90 were discharged and 34 died. There was no censoring in this sample and the last event occurred 82 days after admission.

### Transition rates

Table 1 gives the AIC for each distribution fitted to each transition separately. The AIC indicated that the following models gave the best fit for each transition and were therefore chosen for the “AIC” model:

1. **Transition 1:** Royston-Parmer model with 4 degrees of freedom.

**Table 1:** AIC for each parametric model fitted to each transition separately (to determine the “AIC” model)

Model	Transition 1	Transition 2	Transition 3	Transition 4	Transition 5
Exponential	1229.7	3428.9	1482.3	691.6	328.7
Weibull	1208.6	3363.2	1425.9	692.5	330.0
Gompertz	1230.1	3430.1	1475.6	693.4	328.4
Log-logistic	1193.8	3204.2	1389.6	687.4	328.2
Log-normal	1175.7	3182.6	1374.7	686.6	328.0
Generalised gamma	1141.9	3047.6	1361.0	687.3	325.0
R-P DF=2	1168.1	3163.1	1367.3	687.7	327.1
R-P DF=3	1141.5	3081.4	1367.0	687.9	328.3
R-P DF=4	1136.8	3072.5	1361.0	689.1	328.7
R-P DF=5	1138.5	3070.3	1363.3	690.8	330.3

R-P = Royston-Parmar, DF = Degrees of freedom
2. **Transition 2:** Generalised gamma model.
3. **Transition 3:** Royston-Parmer model with 4 degrees of freedom.
4. **Transition 4:** Log-normal model.
5. **Transition 5:** Generalised gamma model.

**Fig. 2:**
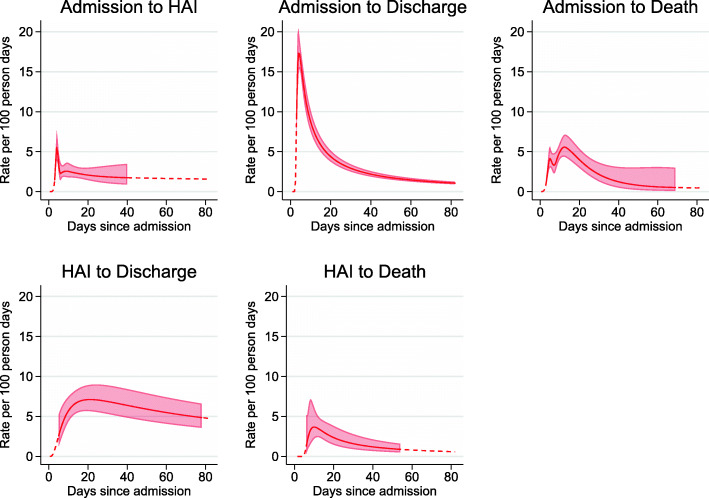
Transition rates from the “AIC” model with 95% confidence intervals (shaded region). Point estimates and confidence intervals were defined from the time of the first event until the last event for each transition (solid lines). The point estimates were extrapolated to cover the interval [0,82] (dashed line)

Figure 2 illustrates the transition rates from the “AIC” model. The point estimates and confidence intervals are shown from the time of the first event until the last event for each transition by a solid line. The corresponding intervals were [3,40],[3,82],[3,69],[5,78] and [6,54] for transitions 1-5, respectively. The point estimates were extrapolated to cover the interval [0,82] with a dashed line. It was evident that the transition rates were not constant over time and transition 2 (admission to discharge without HAI) appeared to deviate most drastically from this assumption. Additional file 3: Figure S1 compares the transition rates from the “AIC” model to the “Exp” model, “RP(4)” model and to non-parametric estimates obtained using the Epanechnikov kernel smoother. The smoothed non-parametric estimates varied depending on the kernel type and bandwidth used, however, in all cases, the transition rates were clearly not constant.

### Transition probabilities

**Fig. 3:**
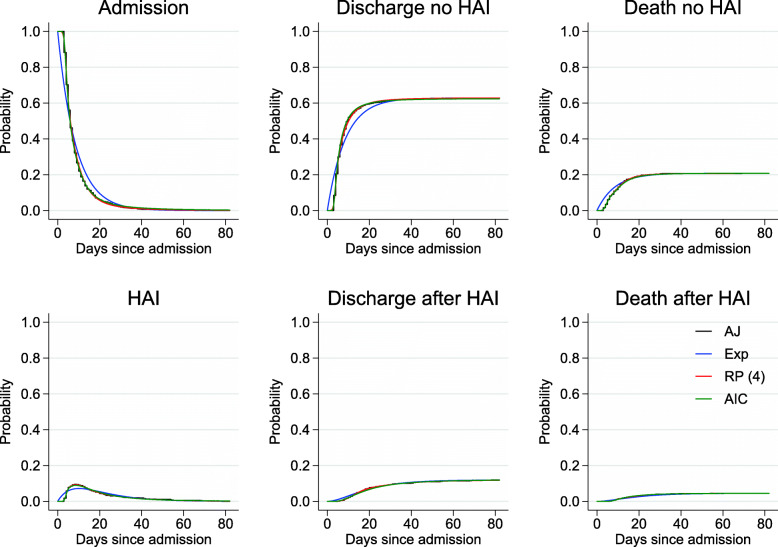
Transition probabilities from state 1 at time 0 to each state for the different approaches: “AJ” (black), “Exp” (blue), “RP(4)” (red) and “AIC” (green). Note that there is considerable overlap between the “RP(4)” and “AIC” estimates

Figure 3 compares the transition probability estimates from the three approaches with empirical estimates (starting in state 1 at time 0). The predictions from the “AIC” and “RP(4)” models had high concordance with the Aalen-Johansen estimates. As von Cube et al. noted [18], despite clear departures from the constant transition rates assumption, the “Exp” model performed well for states 4, 5 and 6 (death without HAI, discharge with HAI and death with HAI). There were some discrepancies with states 1, 2 and 3 (hospital admission without HAI, with HAI and discharge with HAI) up to 30 days after admission. Importantly, the predictions obtained from the “AIC” and “RP(4)” models captured the three day delay in acquiring a HAI (and in fact the delay in experiencing any event, as the minimum event time was 3 days since admission), which the “Exp” model could not capture. Where estimates are shown comparing approaches in the main body of the paper, Additional file 3: Figures S2, S5 and S6 show the corresponding graphs for the “AIC” model with confidence intervals.

Additional file 3: Figure S3 illustrates the transition probabilities conditional on being in state 2 (in hospital with a HAI) by time 3. Predictions from the “AIC” and “RP(4)” models were slightly more consistent with the empirical estimates than those from the “Exp” model (especially in the first 20 days). Additional file 3: Figure S4 is the corresponding graph for the “AIC” model with confidence intervals.

### Attributable mortality and population attributable fraction

About 20-25 days after hospital admission, AM was above 0 for the “AIC” and “RP(4)” models. This suggested that after 25 days, the probability of dying was greater for those with an infection than for those without. AM can be interpreted as, for example: an individual that acquired a HAI by time 10 had a 4.7 percentage point decreased probability of dying by time 10 compared to an individual who did not acquire a HAI. Alternatively, an individual that acquired a HAI by time 30 had a 1.4 percentage point increased probability of dying by time 30. These results were similar for PAF, suggesting that after 25 days the occurrence of a HAI increased the risk of death and therefore the overall probability of dying. PAF can be interpreted as, for example: the proportion of individuals dying by time 10 would have increased by 4.2% if there were no HAIs. Alternatively, the proportion of individuals dying by time 30 would have decreased by 0.9% if there were no HAIs (all predictions from the “AIC” model). Schumacher et al. [23] describes the phenomenon of the AM and PAF initially being lower than 0.

**Fig. 4:**
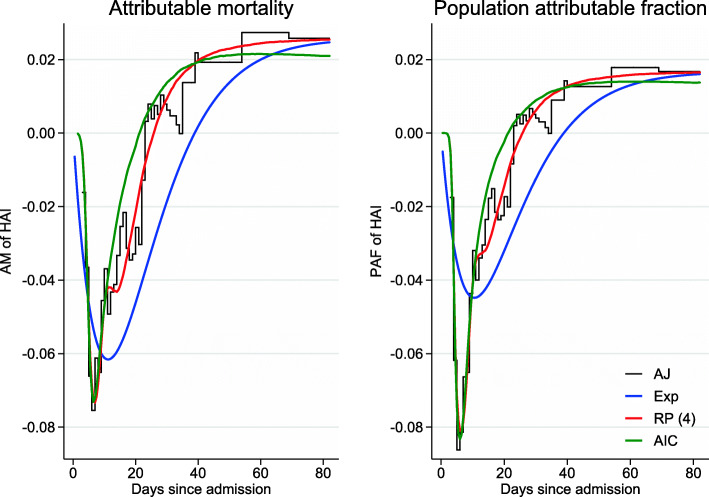
Attributable mortality (AM) and population attributable fraction (PAF) of HAIs for the different approaches: “AJ” (black), “Exp” (blue), “RP(4)” (red) and “AIC” (green)

The relative differences in predictions between the models were greatest for AM and PAF, see Fig. 4 (although the absolute differences were small). The “Exp” model appeared to overestimate (until 15 days after admission) and then underestimate AM and PAF considerably; whereas both “AIC” and “RP(4)” predictions appeared to approximate the empirical estimates well. There was a slight inconsistency with the “AIC” model and the empirical estimates towards the end of the time window, however, this should not be over-interrupted due to the small number of events occurring past 50 days.

### Length of stay

**Fig. 5:**
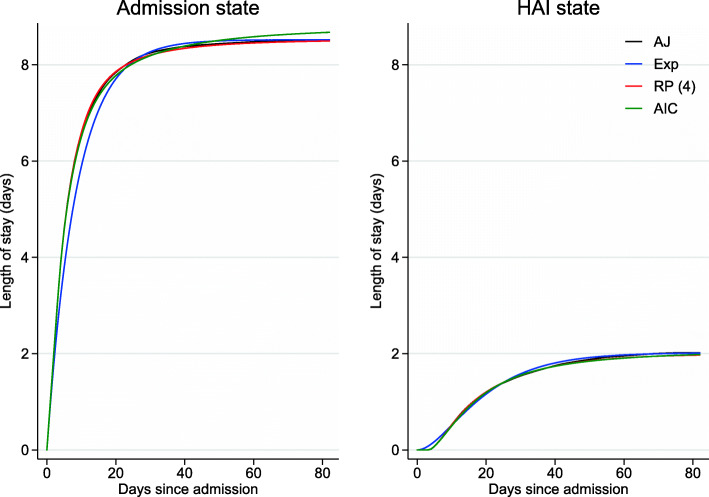
Length of stay in hospital without (state 1, left) and with (state 2, right) a HAI from state 1 at time 0 for the different approaches: “AJ” (black), “Exp” (blue), “RP(4)” (red) and “AIC” (green)

Figure 5 illustrates the restricted expected length of stay for the three models with empirical estimates (starting in state 1 at time 0). The graph can be interpreted as follows: 82 days since hospital admission, on average a patient spent 8.68 (95*%* CI 8.04,9.39) days in hospital without a HAI and 1.98 (95*%* CI 1.53,2.58) days in hospital with a HAI (estimates taken from the “AIC” model). The empirical estimates were slightly better approximated by the “AIC” and “RP(4)” models (especially in the first 20 days). The confidence intervals for total hospital stay for the “AIC” model are shown in Additional file 3: Figure S7.

**Fig. 6:**
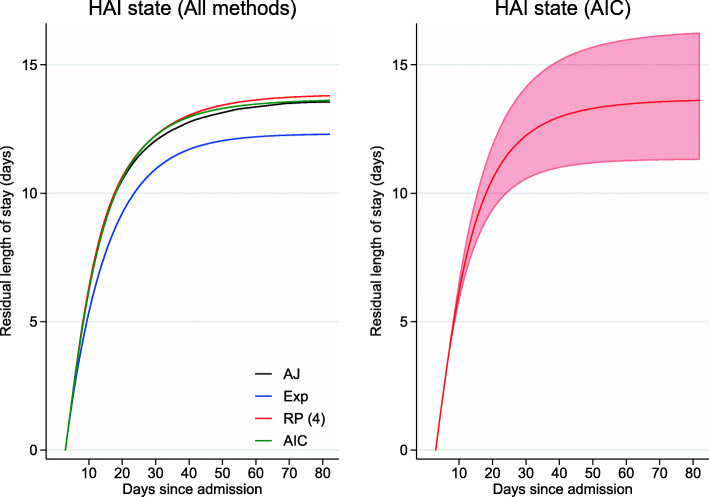
Residual length of stay in hospital with a HAI (state 2) from state 2 at time 3. In the left panel, for the different approaches:“AJ” (black), “Exp” (blue), “RP(4)” (red) and “AIC” (green). In the right panel, for the “AIC” model (solid line) with 95% confidence intervals (shaded region)

Figure 6 illustrates the residual, restricted expected length of stay, conditional on being in state 2 (in hospital with a HAI) by time 3. Between the interval [3,82] days, a patient would have spent on average 13.61 (95*%* CI 11.30,16.26) days in hospital with a HAI, given they had a HAI and were in hospital by time 3 (estimates taken from the “AIC” model). While predictions obtained from the “AIC” and “RP(4)” models were consistent with empirical results, Fig. 6 shows a large discrepancy between the latter and the “Exp” model. In the context of health economics, such differences could be non-trivial when translated into costs.

## Discussion

Assuming constant transition rates in a multi-state model can facilitate a basic understanding of the data and this approach has been well demonstrated by von Cube et al. [18]. However, this assumption may not always be plausible and, as a result, predictions may be misspecified. In the example shown, despite the transition rates not being constant (Fig. 2), the transition probabilities from the “Exp” approach were similar to the Aalen-Johansen estimates. However, this was not the case for some functions of the transition probabilities, for example, AM and PAF (Fig. 4). In addition, predictions from the “Exp” model starting in state 2 at time 3 had poorer concordance with the non-parametric estimates (Additional file 3: Figure S3 and Fig. 6).

This paper compared the “Exp” model to two parametric approaches, where predictions from the latter were obtained from the fitted model via a general simulation algorithm. The “AIC” and “RP(4)” predictions were more consistent with the Aalen-Johansen estimates than the “Exp” model for all metrics. The greatest improvements were seen in AM and PAF and when considering delayed entry (predictions starting from state 2 at time 3).

As with any parametric approach, assumptions need to be made regarding the most appropriate distribution for each transition. A balance needs to be sought in terms of parsimony and sufficient parameters to appropriately capture the hazard shapes. This work has highlighted the challenges of model selection as the “AIC” model (i.e. the collection of the best fitting distributions for each transition in terms of AIC) did not always have better concordance with the non-parametric estimates compared to the reference “RP(4)” model. Regardless of approach, we would always recommend sensitivity analyses around the assumptions of the baseline hazard. It is important to note that both approaches still performed better than the “Exp” model. For this data example, a conditional parametric model would have been more appropriate for any transitions that could not have happened before day 3 (by definition or design of the study). We chose not to consider a conditional model to be consistent with, and allow easier comparison with, the motivating paper by von Cube et al. [18].

In addition to being able to model the transitions with a range of parametric distributions, the general simulation algorithm has other advantages. It easily lends itself to extended predictions, such as length of stay, the probability of ever visiting a state and disease specific quantities, such as AM and PAF. Uncertainties can easily be obtained, which can facilitate a more comprehensive understanding of the data. There is also great flexibility available when modelling covariate effects, including time-dependent effects [12]. The approach generalises to more complex multi-state models, i.e. models with a greater number of states, greater number of transitions and backward transitions. It can also be applied to non-Markov models, unlike methods that rely on solving the Kolmogorov forward equations to obtain predictions.

A disadvantage of the general simulation algorithm is computational time. Although point estimates can be obtained relatively quickly, confidence intervals can require a considerable amount of time, especially in the case of more complicated user-defined functions. A balance between computational time and Monte Carlo error is therefore needed when choosing *N* (number of simulations) and *M* (number of repetitions for confidence intervals). One possible alternative would be to use a hybrid approach when calculating predictions, where the transition rates obtained through parametric methods are substituted into the non-parametric Aalen-Johansen estimator [15, 30]. This approach would greatly decrease computational time, however, is only applicable to Markov models.

## Conclusion

We conclude that methods and software are readily available for obtaining predictions from multi-state models that do not assume constant transition rates. The multistate package in Stata facilitates a range of predictions with confidence intervals obtained from a fitted multi-state model via a general simulation algorithm. We agree that assuming constant transition rates can provide a quick, basic understanding of the data; however, we recommend a more sophisticated parametric approach for a comprehensive understanding that includes uncertainty.

## Supplementary Information

**Additional file 1** Stata code for predictions. A Word document (.docx) with Stata code to analyse the HAI data. It formats the los.data dataset (once imported from the R package etm), fits the parametric models and obtains the predictions from the different approaches (Aalen-Johansen estimates, “Exp” model, “AIC” model and “RP(4)” model). The predictions include all those calculated in this paper: transition probabilities, AM and PAF and expected length of stay.

**Additional file 2** Further details of the analysis approaches. A PDF giving details about how the Aalen-Johansen estimates were calculated and showing the functional forms of the transition rates for the three parametric approaches.

**Additional file 3** Additional figures. A PDF including supplementary figures from the HAI data analysis.

## Data Availability

The dataset analysed is publicly available in the Comprehensive R Archive Network package etm [24]. Stata code is available in Additional file 1 of this paper.

## Abbreviations

AIC: Akaike information criterion
AM: Attributable mortality
HAI: Hospital acquired infection
PAF: Population attributable fraction
