# Supplementary material for: Relaxing the assumption of constant transition rates in a multi-state model in hospital epidemiology
Source: BMC Med Res Methodol. 2021 Jan 11;21:16. doi: 10.1186/s12874-020-01192-8 (PMC7798316; doi:10.1186/s12874-020-01192-8)
Supplement: Supplementary file 1 — Additional file 1 Stata code for predictions. A Word document (.docx) with Stata code to analyse the HAI data. It formats the los.data dataset (once imported from the R package etm), fits the parametric models and obtains the predictions from the different approaches (Aalen-Johansen estimates, “Exp” model, “AIC” model and “RP(4)” model). The predictions include all those calculated in this paper: transition probabilities, AM and PAF and expected length of stay. [file 12874_2020_1192_MOESM1_ESM.docx]

**Additional File 1: Stata code for predictions**

// Install multistate package (and other packages if needed)

ssc install multistate

ssc install merlin

// ssc install moremata

// Export the data from R into a csv file (in your local directory), R code:

// install.packages("etm")

// data(los.data, package="etm")

// write.csv(los.data, file = "los.csv", row.names = F)

// Import the data into Stata and format

import delimited using los, clear asdouble

rename (j01 j02 j03 j12 j13) (t12 t13 t14 t25 t26)

local var_list t12 t13 t14 t25 t26

forvalues i=1/5 {

// Change event times to numeric

local var `: word `i' of `var_list''

replace `var' = "" if `var' == "Inf"

destring `var', replace

// Create an indicator to say whether the patient experienced that transition

gen status`i' = 0

local var `: word `i' of `var_list''

replace status`i' = 1 if `var' != .

}

// Fill in same event times for transitions that the patient was at risk at

foreach var in t12 t13 t14 {

replace `var' = max(t12, t13, t14)

}

foreach var in t25 t26 {

replace `var' = max(t25, t26)

}

// Transition matrix

mat tmat = (.,1,2,3,.,.\.,.,.,.,4,5\.,.,.,.,.,.\.,.,.,.,.,.\.,.,.,.,.,.\.,.,.,.,.,.)

mat list tmat

// Prepare the data into stacked format

msset, id(admid) states(status1 status2 status3 status4 status5) times(t12 t13 t14 t25 t26) transmat(tmat)

// Declare data as survival data

stset _stop, failure(_status) enter(_start)

// Create time variables for the predictions starting at day 0 and day 3 at half day time points

range timevar0 0 82 165

range timevar3 3 82 159

// Picking the AIC model

forvalues i = 1/5 {

display "Transition `i'"

// Exponential

merlin (_stop if _trans == `i', family(exponential, failure(_status) ltruncated(_start)))

estimates store m`i'_exp

// Weibull

merlin (_stop if _trans == `i', family(weibull, failure(_status) ltruncated(_start)))

estimates store m`i'_weib

// Gompertz

merlin (_stop if _trans == `i', family(gompertz, failure(_status) ltruncated(_start)))

estimates store m`i'_gom

// Log logistic

merlin (_stop if _trans == `i', family(loglogistic, failure(_status) ltruncated(_start)))

estimates store m`i'_logl

// Log normal

merlin (_stop if _trans == `i', family(lognormal, failure(_status) ltruncated(_start)))

estimates store m`i'_logn

// Generalised gamma

merlin (_stop if _trans == `i', family(ggamma, failure(_status) ltruncated(_start)))

estimates store m`i'_ggam

// Royston Parmar models

forvalues j=2/5 {

merlin (_stop if _trans == `i', family(rp, df(`j') failure(_status) ltruncated(_start)))

estimates store m`i'_rp`j'

}

}

// Pick the model with lowest AIC for each transition

forvalues i = 1/5 {

di "Transition `i'"

qui count if _trans == `i' & _d == 1

estimates stat m`i'*, n(`r(N)')

}

estimates clear

// Transition rates

// Exp model

forvalues i = 1/5 {

merlin (_stop if _trans == `i', family(exponential, failure(_status) ltruncated(_start)))

predict ehaz`i', hazard

}

// RP(4) model

forvalues i = 1/5 {

merlin (_stop if _trans == `i', family(rp, df(4) failure(_status) ltruncated(_start)))

estimates store m_rp`i'

predict rphaz`i', hazard timevar(timevar0)

}

// AIC model

merlin (_stop if _trans == 1, family(rp, df(4) failure(_status) ltruncated(_start)))

estimates store m_f1

predict fhaz1, ci hazard timevar(timevar0)

merlin (_stop if _trans == 2, family(ggamma, failure(_status) ltruncated(_start)))

estimates store m_f2

predict fhaz2, ci hazard timevar(timevar0)

merlin (_stop if _trans == 3, family(rp, df(4) failure(_status) ltruncated(_start)))

estimates store m_f3

predict fhaz3, ci hazard timevar(timevar0)

merlin (_stop if _trans == 4, family(lognormal, failure(_status) ltruncated(_start)))

estimates store m_f4

predict fhaz4, ci hazard timevar(timevar0)

merlin (_stop if _trans == 5, family(ggamma, failure(_status) ltruncated(_start) )

estimates store m_f5

predict fhaz5, ci hazard timevar(timevar0)

// Transition probabilities

// From time 0: AJ estimates

msaj, transm(tmat)

rename (P_AJ_*) (ajprob*)

// From time 0: Exp model

merlin (_stop _trans1 _trans2 _trans3 _trans4 _trans5, ///

family(exponential, failure(_status) ltruncated(_start)) noconstant)

global haz12 = exp([_cmp_1_1_1]_b[_cons])

global haz13 = exp([_cmp_1_2_1]_b[_cons])

global haz14 = exp([_cmp_1_3_1]_b[_cons])

global haz25 = exp([_cmp_1_4_1]_b[_cons])

global haz26 = exp([_cmp_1_5_1]_b[_cons])

global haz1=$haz12 + $haz13 + $haz14

global haz2=$haz25 + $haz26

gen double eprob1= exp(-1* $haz1 *timevar0)

gen double eprob2= ($haz12 /($haz2 -$haz1 ))*(exp(-1*$haz1 *timevar0)-exp(-1*$haz2 *timevar0))

gen double eprob3= ($haz13 /$haz1 )*(1-exp(-1*$haz1 *timevar0))

gen double eprob4= ($haz14 /$haz1 )*(1-exp(-1*$haz1 *timevar0))

gen double eprob5= ($haz12 *$haz25 )/($haz1 *$haz2 ) - ///

(($haz12 *$haz25 )/($haz1 *($haz2 -$haz1 )))*exp(-1*$haz1 *timevar0) + ///

(($haz12 *$haz25 )/($haz2 *($haz2 -$haz1 )))*exp(-1*$haz2 *timevar0)

gen double eprob6= ($haz12 *$haz26 )/($haz1 *$haz2 ) - ///

(($haz12 *$haz26 )/($haz1 *($haz2 -$haz1 )))*exp(-1*$haz1 *timevar0) + ///

(($haz12 *$haz26 )/($haz2 *($haz2 -$haz1 )))*exp(-1*$haz2 *timevar0)

// From time 0: RP(4) model

predictms, transm(tmat) timevar(timevar0) models(m_rp1 m_rp2 m_rp3 m_rp4 m_rp5) ///

seed(3819407) n(1000000) probability latent

rename (_prob_at1_1_*) (rpprob*)

// From time 0: AIC model

predictms, transm(tmat) timevar(timevar0) models(m_f1 m_f2 m_f3 m_f4 m_f5) ///

seed(3819407) n(1000000) probability latent

rename (_prob_at1_1_*) (fprob*)

// From time 0: AIC model - CIs

predictms, transm(tmat) timevar(timevar0) models(m_f1 m_f2 m_f3 m_f4 m_f5) ///

seed(3819407) n(100000) ci m(500) probability latent

rename (_prob_at1_1_*lci) (fprob*lci)

rename (_prob_at1_1_*uci) (fprob*uci)

// From time 3: AJ estimates

msaj, transm(tmat) from(2) ltruncated(3)

rename (P_AJ_*) (ajprob_from2_*)

// From time 3: Exp model

gen double eprob_from2_2 = exp(-1*$haz2 *(timevar3-3))

gen double eprob_from2_5 = ($haz25 /$haz2 )*(1-exp(-1*$haz2 *(timevar3-3)))

gen double eprob_from2_6 = ($haz26 /$haz2 )*(1-exp(-1*$haz2 *(timevar3-3)))

// From time 3: RP(4) model

predictms, transm(tmat) timevar(timevar3) models(m_rp1 m_rp2 m_rp3 m_rp4 m_rp5) ///

seed(3819407) n(1000000) from(2) probability latent ltruncated(3)

rename (_prob_at1_2_*) (rpprob_from2_*)

// From time 3: AIC model

predictms, transm(tmat) timevar(timevar3) models(m_f1 m_f2 m_f3 m_f4 m_f5) ///

seed(3819407) n(1000000) from(2) probability latent ltruncated(3)

rename (_prob_at1_2_*) (fprob_from2_*)

// From time 3: AIC model - CIs

predictms, transm(tmat) timevar(timevar3) models(m_f1 m_f2 m_f3 m_f4 m_f5) ///

seed(3819407) n(100000) from(2) ci m(500) probability latent ltruncated(3)

rename (_prob_at1_2_*lci) (fprob_from2_*lci)

rename (_prob_at1_2_*uci) (fprob_from2_*uci)

// Attributable mortality (AM) & Population Attributable Fraction (PAF)

// AJ estimates

gen double ajPdead=ajprob4+ajprob6

gen double ajPdead_HAI0=ajprob4/(ajprob1+ajprob3+ajprob4)

gen double ajPdead_HAI1=ajprob6/(ajprob2+ajprob5+ajprob6)

gen double ajAM=ajPdead_HAI1-ajPdead_HAI0

gen double ajPAF=(ajPdead-ajPdead_HAI0)/ajPdead

// Exp model

gen double ePdead=eprob4+eprob6

gen double ePdead_HAI0=eprob4/(eprob1+eprob3+eprob4)

gen double ePdead_HAI1=eprob6/(eprob2+eprob5+eprob6)

gen double eAM=ePdead_HAI1-ePdead_HAI0

gen double ePAF=(ePdead-ePdead_HAI0)/ePdead

mata:

real matrix GenRes(M)

{

//Extract transition probabilities

p1 = ms_user_prob(M,1)

p2 = ms_user_prob(M,2)

p3 = ms_user_prob(M,3)

p4 = ms_user_prob(M,4)

p5 = ms_user_prob(M,5)

p6 = ms_user_prob(M,6)

// Mortality

Pdead = p4+p6

Pdead_HAI0 = p4:/(p1:+p3:+p4)

Pdead_HAI1 = p6:/(p2:+p5:+p6)

AM = Pdead_HAI1 :- Pdead_HAI0

PAF = (Pdead :- Pdead_HAI0):/Pdead

// Return results

return(AM,PAF)

}

end

// RP(4) model

predictms, transm(tmat) timevar(timevar0) models(m_rp1 m_rp2 m_rp3 m_rp4 m_rp5) ///

seed(3819407) n(1000000) userfunction(GenRes) probability latent

rename (_user_at1_1_1) (rpAM)

rename (_user_at1_1_2) (rpPAF)

// AIC model

predictms, transm(tmat) timevar(timevar0) models(m_f1 m_f2 m_f3 m_f4 m_f5) ///

seed(3819407) n(1000000) userfunction(GenRes) probability latent

rename (_user_at1_1_1) (fAM)

rename (_user_at1_1_2) (fPAF)

// AIC model - CIs

predictms, transm(tmat) timevar(timevar0) models(m_f1 m_f2 m_f3 m_f4 m_f5) ///

seed(3819407) n(100000) userfunction(GenRes) ci m(500) probability latent

rename (_user_at1_1_1_lci) (fAM_lci)

rename (_user_at1_1_1_uci) (fAM_uci)

rename (_user_at1_1_2_lci) (fPAF_lci)

rename (_user_at1_1_2_uci) (fPAF_uci)

// Length of stay

// From time 0: AJ estimates

msaj, transm(tmat) los

rename (LOS_AJ_*) (ajlos*)

drop P_AJ_*

// From time 0: Exp model

gen double elos1=(1/$haz1 )*(1-exp(-1*$haz1 * timevar0))

gen double elos2=($haz12 /($haz2 - $haz1 ))* ///

((exp(-1*$haz2 *timevar0)/$haz2 ) - ///

(exp(-1*$haz1 *timevar0)/ $haz1 ) + ///

(1/$haz1 ) - 1/$haz2 )

// From time 0: RP(4) model

predictms, transm(tmat) timevar(timevar0) models(m_rp1 m_rp2 m_rp3 m_rp4 m_rp5) ///

seed(3819407) n(1000000) los latent

rename (_los_at1_1_*) (rplos*)

// From time 0: AIC model

predictms, transm(tmat) timevar(timevar0) models(m_f1 m_f2 m_f3 m_f4 m_f5) ///

seed(3819407) n(1000000) los latent

rename (_los_at1_1_*) (flos*)

// From time 0: AIC model - CIs

predictms, transm(tmat) timevar(timevar0) models(m_f1 m_f2 m_f3 m_f4 m_f5) ///

seed(3819407) n(100000) los ci m(500) latent

rename (_los_at1_1_*lci) (flos*lci)

rename (_los_at1_1_*uci) (flos*uci)

// From time 3: AJ estimates

msaj, transm(tmat) los from(2) ltruncated(3)

rename (LOS_AJ_*) (ajlos_from2_*)

// From time 3: Exp model

gen double elos_from2_2 =(1/$haz2 )*(1-exp(-1*$haz2 * (timevar3-3)))

// From time 3: RP(4) model

predictms, transm(tmat) timevar(timevar3) models(m_rp1 m_rp2 m_rp3 m_rp4 m_rp5) ///

seed(3819407) n(1000000) los from(2) ltruncated(3) latent

rename (_los_at1_2_*) (rplos_from2_*)

// From time 3: AIC model

predictms, transm(tmat) timevar(timevar3) models(m_f1 m_f2 m_f3 m_f4 m_f5) ///

seed(3819407) n(1000000) los from(2) ltruncated(3) latent

rename (_los_at1_2_*) (flos_from2_*)

// From time 3: AIC model - CIs

predictms, transm(tmat) timevar(timevar3) models(m_f1 m_f2 m_f3 m_f4 m_f5) ///

seed(3819407) n(100000) los from(2) ci m(500) ltruncated(3) latent

rename (_los_at1_2_*lci) (flos_from2_*lci)

rename (_los_at1_2_*uci) (flos_from2_*uci)

// Total stay in hospital

mata:

real matrix Lhosp(M)

{

// Extract transition probabilities

los1 = ms_user_los(M,1)

los2 = ms_user_los(M,2)

// Hospital states

total_los = los1+los2

// Return results

return(total_los)

}

end

// AIC model

predictms, transm(tmat) timevar(timevar0) models(m_f1 m_f2 m_f3 m_f4 m_f5) ///

seed(3819407) n(1000000) userfunction(Lhosp) los latent

rename (_user_at1_1_1*) (flos_hosp*)

// AIC model - CIs

predictms, transm(tmat) timevar(timevar0) models(m_f1 m_f2 m_f3 m_f4 m_f5) ///

seed(3819407) n(100000) userfunction(Lhosp) los ci m(500) latent

rename (_user_at1_1_1*lci) (flos_hosp*lci)

rename (_user_at1_1_1*uci) (flos_hosp*uci)
