# Supplementary material for: Relaxing the assumption of constant transition rates in a multi-state model in hospital epidemiology
Source: BMC Med Res Methodol. 2021 Jan 11;21:16. doi: 10.1186/s12874-020-01192-8 (PMC7798316; doi:10.1186/s12874-020-01192-8)
Supplement: Supplementary file 2 — Additional file 2 Further details of the analysis approaches. A PDF giving details about how the Aalen-Johansen estimates were calculated and showing the functional forms of the transition rates for the three parametric approaches. [file 12874_2020_1192_MOESM2_ESM.pdf]

Additional File 2: Further Details of the Analysis Approaches

Aalen-Johansen Estimator

The Aalen-Johansen estimator generalises the Kaplan-Meier estimator to Markov multistate processes. It was proposed by Aalen and Johansen [1] and has been discussed in detail by Andersen et al [2]. Following Allignol et al [3], let $N_{ab}(t)$ be the number of direct transitions $a \to b$ occurring up to time t and $Y_a(t)$ be the number of individuals in state a at the time immediately before t, i.e. the number of individuals at risk of the transition $a \to b$. The matrix of cumulative transition hazards $\mathbf{A}(t)$ can be estimated by the Nelson-Aalen estimator [2]:

$$\hat{A}_{ab}(t) = \int_0^t \frac{dN_{ab}(u)}{Y_a(u)} du \qquad a \neq b$$
$$\hat{A}_{aa}(t) = -\sum_{b \neq a} \hat{A}_{ab}(t)$$

The Aalen-Johansen estimator of the transition probabilities is then:

$$\hat{\mathbf{P}}(s,t) = \prod_{s < t_k \leq t} \Big(\mathbf{I} + \triangle\hat{\mathbf{A}}(t_k)\Big)$$

Where $\triangle\hat{A}_{ab}(t_k)$ $a \neq b$ is the number of observed direct $a \to b$ transitions divided by the number of individuals in state a at the time immediately before t_k. The diagonal entries $\triangle\hat{A}_{aa}(t_k)$ are such that the row equals 0.

The Aalen-Johansen estimator is a matrix of step-functions, changing only at the times when an event is observed. Expected length of stay can then be easily calculated as a summation of rectangles.

Exponential Model

For transitions $i = 1, ..5$, the transition rates $h_i(t)$ for the exponential model are a non-negative constant $\lambda_i \geq 0$:

$$h_i(t) = \lambda_i$$

Royston-Parmar Model (4 degrees of freedom)

Royston-Parmar models utilise restricted cubic splines to model the effect of time on the log cumulative hazard $\ln\{H_i(t)\}$ scale. A Royston-Parmar model with K knots can be fitted by creating $K-1$ derived variables [4]. For models with $K = 5$, there will be 4 derived variables for each transition i: z_{ij}, $j = 1, ...4$ and 5 knots for each transition i: k_{il}, $l = 1, ...5$. The equation for $\ln\{H_i(t)\}$ and all necessary components are [4]:

$$\ln\{H_i(t)\} = \gamma_{i0} + \gamma_{i1}z_{i1} + \gamma_{i2}z_{i2} + \gamma_{i3}z_{i3} + \gamma_{i4}z_{i4}$$
$$z_{i1} = \ln(t)$$
$$z_{ij} = (\ln(t) - k_{ij})^3_+ - \phi_{ij}(\ln(t) - k_{i1})^3_+ - (1 - \phi_{ij})(\ln(t) - k_{i5})^3_+ \quad j = 2, 3, 4$$
$$\phi_{ij} = (k_{i5} - k_{ij})/(k_{i5} - k_{i1})$$

Where γ_{ij} are the parameters to be estimated from the data. The internal knot locations k_{i2}, k_{i3} and k_{i4} were chosen as the 25th, 50th and 75th centiles of the distribution of uncensored log event times for transition i, respectively [4]. k_{i1} and k_{i5} are the boundary knots, located at the minimum and maximum of the uncensored log event times [4]. The model described here has 5 parameters: the 4 derived variables and the constant term. When fitting this model in

Stata using merlin or stpm2, the user would specify 4 degrees of freedom, as the intercept is included by default. For consistency with the programming, we have named this model "RP(4)" (Royston-Parmar with 4 degrees of freedom) in the main text.

The transition rates involve the derivative of the cubic spline functions. See Royston and Parmar [5] and Lambert and Royston [4] for more details on these models.

AIC Model

The "AIC" model involved choosing the distribution with the lowest AIC for each transition. The candidate models were: exponential, Weibull, Gompertz, log-logistic, log-normal, generalised gamma and Royston-Parmar models with 2 to 5 degrees of freedom. The AIC results are given in the Results section under the subsection Transition Rates. The chosen distributions for each transition are repeated here:

1. **Transition 1:** Royston-Parmer model with 4 degrees of freedom.
2. **Transition 2:** Generalised gamma model.
3. **Transition 3:** Royston-Parmer model with 4 degrees of freedom.
4. **Transition 4:** Log-normal model.
5. **Transition 5:** Generalised gamma model.

Parametrisation of the generalised gamma and log-normal distribution are given below. The parameters to be estimated are specific to each transition i, but the subscript has been dropped for easier viewing.

Generalised Gamma

The parametrisation follows the documentation in Stata for streg [6]. The transition rate is defined as $h(t) = f(t)/S(t)$, where the three parametrised gamma density and survivor functions are defined as:

$$f(t) = \begin{cases} \dfrac{\gamma^\gamma}{\sigma t\sqrt{\gamma}\Gamma(\gamma)}\exp(z\sqrt{\gamma} - u) & \kappa \neq 0 \\ \dfrac{1}{\sigma t\sqrt{2\pi}}\exp(-z^2/2) & \kappa = 0 \end{cases}$$

$$S(t) = \begin{cases} 1 - I(\gamma, u) & \kappa > 0 \\ 1 - \Phi(z) & \kappa = 0 \\ I(\gamma, u) & \kappa < 0 \end{cases}$$

Where $\gamma = |\kappa|^{-2}$, $z = \text{sign}(\kappa)\{\ln(t) - \mu\}/\sigma$, $u = \gamma\exp(|\kappa|z)$, $\Phi(.)$ is the standard normal cumulative distribution function and $I(a, x)$ is the incomplete gamma function. The parameter μ and ancillary parameters κ and σ are to be estimated from the data. For more details see the help file for streg [6].

Log-Normal

The parametrisation follows the documentation in `Stata` for `streg` [6] and also that of Royston [7]. The transition rates are defined as $h(t) = f(t)/S(t)$:

$$f(t) = \frac{1}{t\sigma\sqrt{2\pi}}\exp\left(-\frac{1}{2}\left[\frac{\ln(t)-\mu}{\sigma}\right]^2\right)$$
$$S(t) = 1 - \Phi\left(\frac{\ln(t)-\mu}{\sigma}\right)$$

Where $\Phi(.)$ is the standard normal cumulative distribution function. μ is the location parameter and σ^2 is the variance of random variable T. For more details see Royston [7] or the help file for `streg` [6].

References

[1] Aalen OO, Johansen S. An empirical transition matrix for non-homogeneous Markov chains based on censored observations. Scand J Stat. 1978;p. 141–150.

[2] Andersen PK, Borgan O, Gill RD, Keiding N. Statistical models based on counting processes. New York: Springer-Verlag; 1993.

[3] Allignol A, Schumacher M, Beyersmann J. Empirical transition matrix of multi-state models: The etm package. J Stat Softw. 2011;38(4):1–15.

[4] Lambert PC, Royston P. Further development of flexible parametric models for survival analysis. Stata J. 2009;9(2):265–290.

[5] Royston P, Parmar MK. Flexible parametric proportional-hazards and proportional-odds models for censored survival data, with application to prognostic modelling and estimation of treatment effects. Stat Med. 2002;21(15):2175–2197.

[6] StataCorp. 2019. Stata Statistical Software: Release 16. College Station, TX: StataCorp LLC;.

[7] Royston P. The lognormal distribution as a model for survival tme in cancer, with an emphasis on prognostic factors. Stat Neerl. 2001;55(1):89–104.
