# Supplementary material for: Relaxing the assumption of constant transition rates in a multi-state model in hospital epidemiology
Source: BMC Med Res Methodol. 2021 Jan 11;21:16. doi: 10.1186/s12874-020-01192-8 (PMC7798316; doi:10.1186/s12874-020-01192-8)
Supplement: Supplementary file 3 — Additional file 3 Additional figures. A PDF including supplementary figures from the HAI data analysis. [file 12874_2020_1192_MOESM3_ESM.pdf]

Additional File 3: Additional Figures

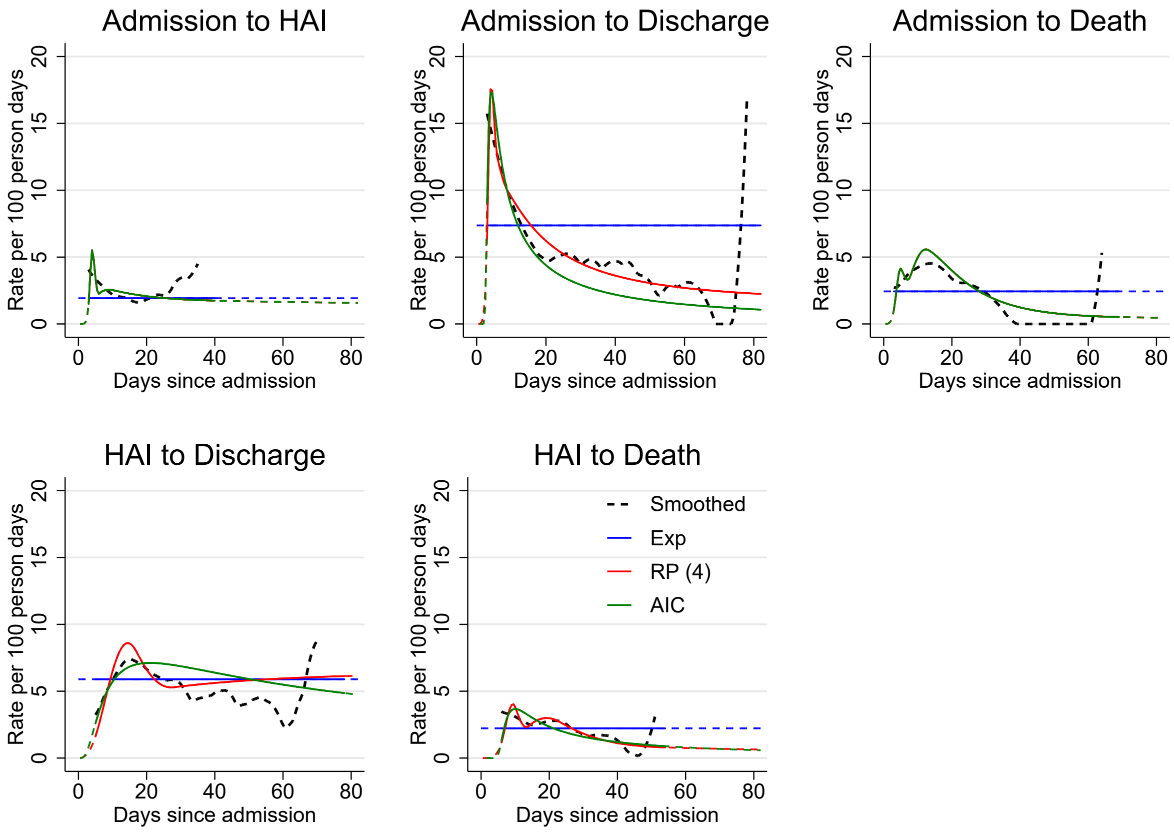

Supplementary Figure 1: Transition rates for the different approaches: Smoothed (dashed black), "Exp" (blue), "RP(4)" (red) and "AIC" (green). The Epanechnikov kernel was used for the smoothed non-parametric estimates. Estimates from the parametric approaches were defined from the time of the first event until the last event for each transition by a solid line and were extrapolated to cover the interval [0, 82] by a dashed line. The smoothed non-parametric estimates were truncated up to 8 days before the last event, as we did not believe the transition rates increased so drastically at the time of the last event

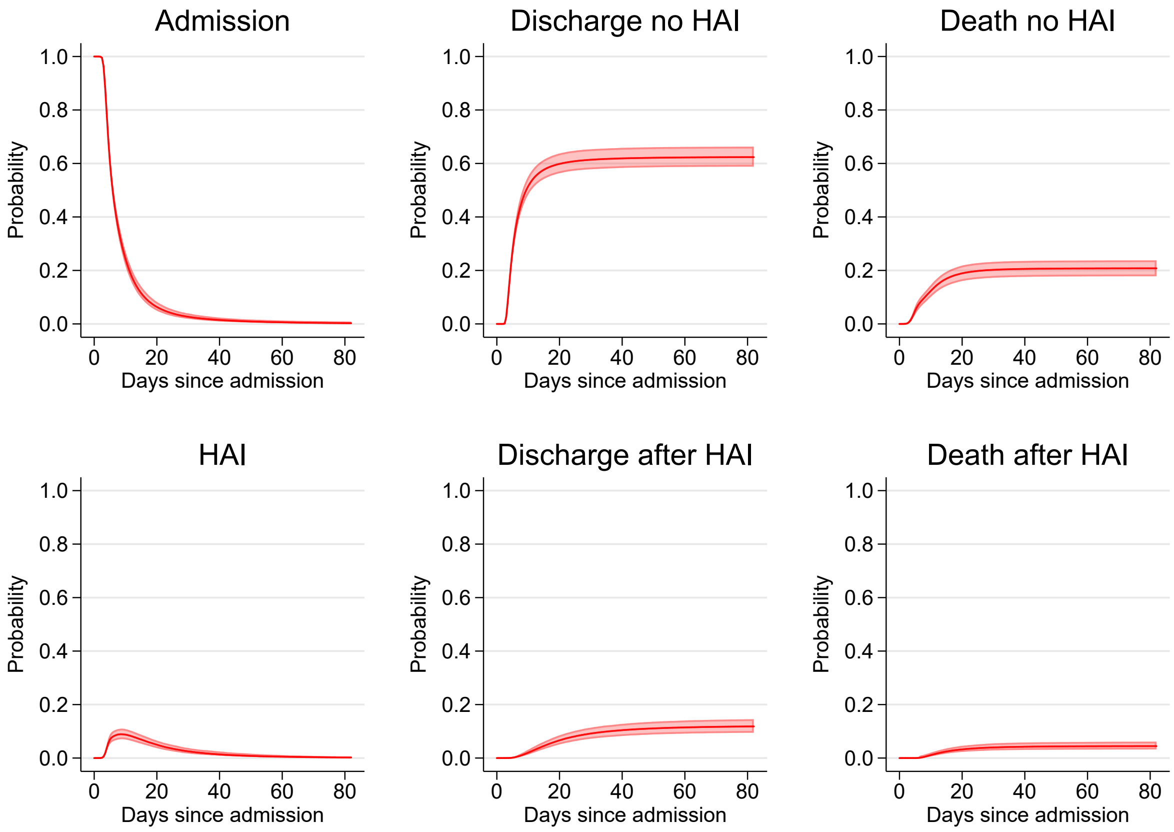

Supplementary Figure 2: Transition probabilities from state 1 at time 0 to each state for the "AIC" model (solid line) with 95% confidence intervals (shaded region)

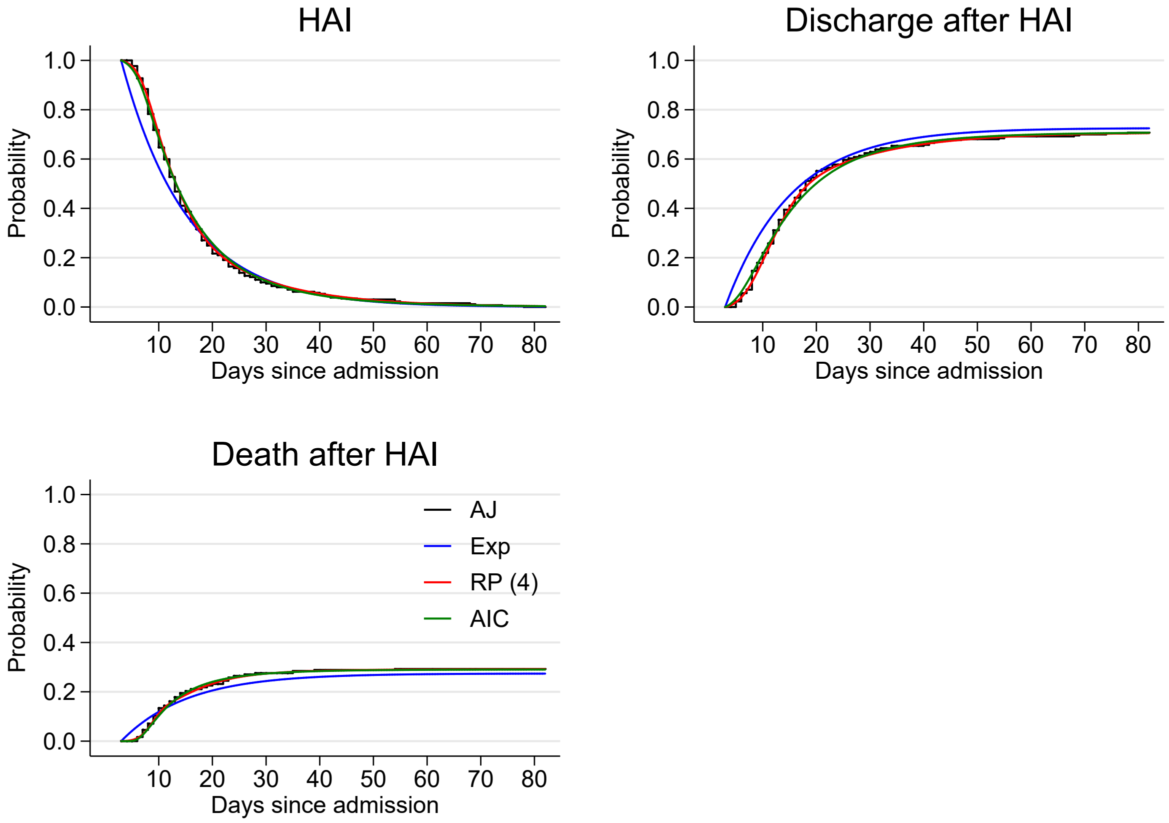

Supplementary Figure 3: Transition probabilities from state 2 at time 3 to the relevant states for the different approaches: "AJ" (black), "Exp" (blue), "RP(4)" (red) and "AIC" (green)

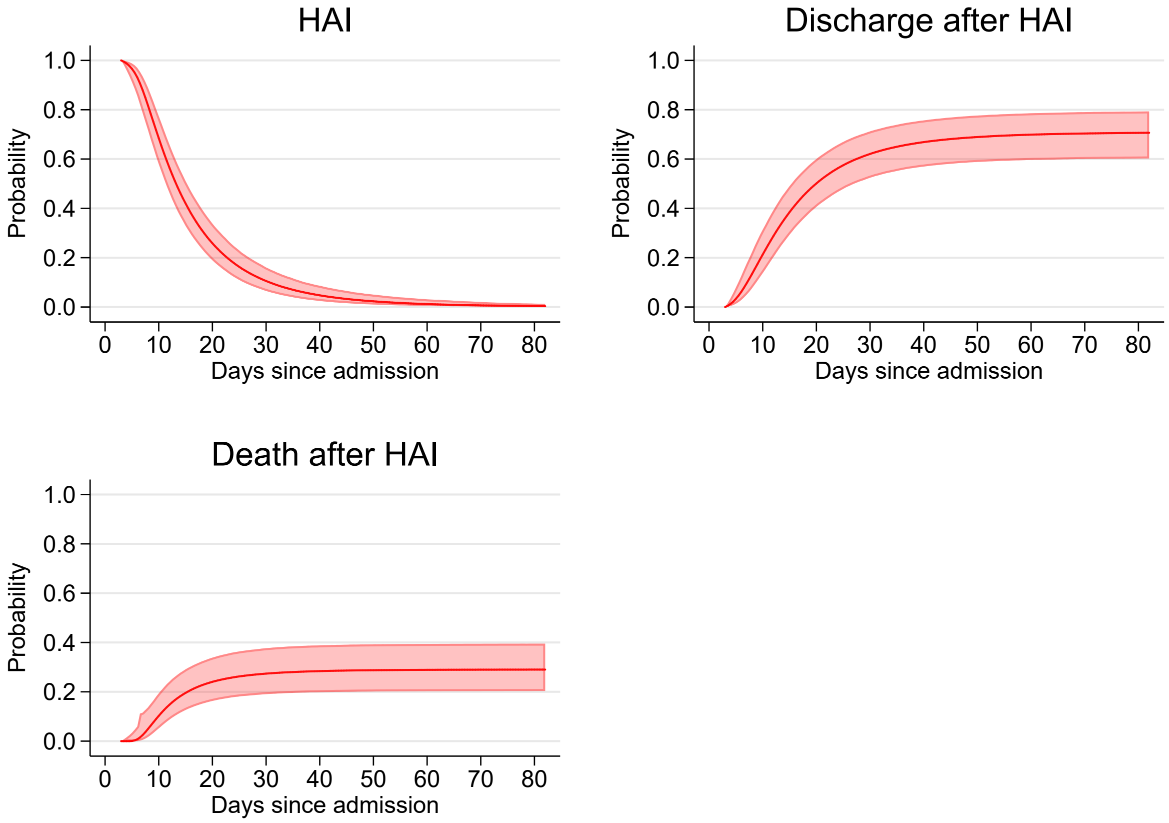

Supplementary Figure 4: Transition probabilities from state 2 at time 3 to the relevant states for the "AIC" model (solid line) with 95% confidence intervals (shaded region)

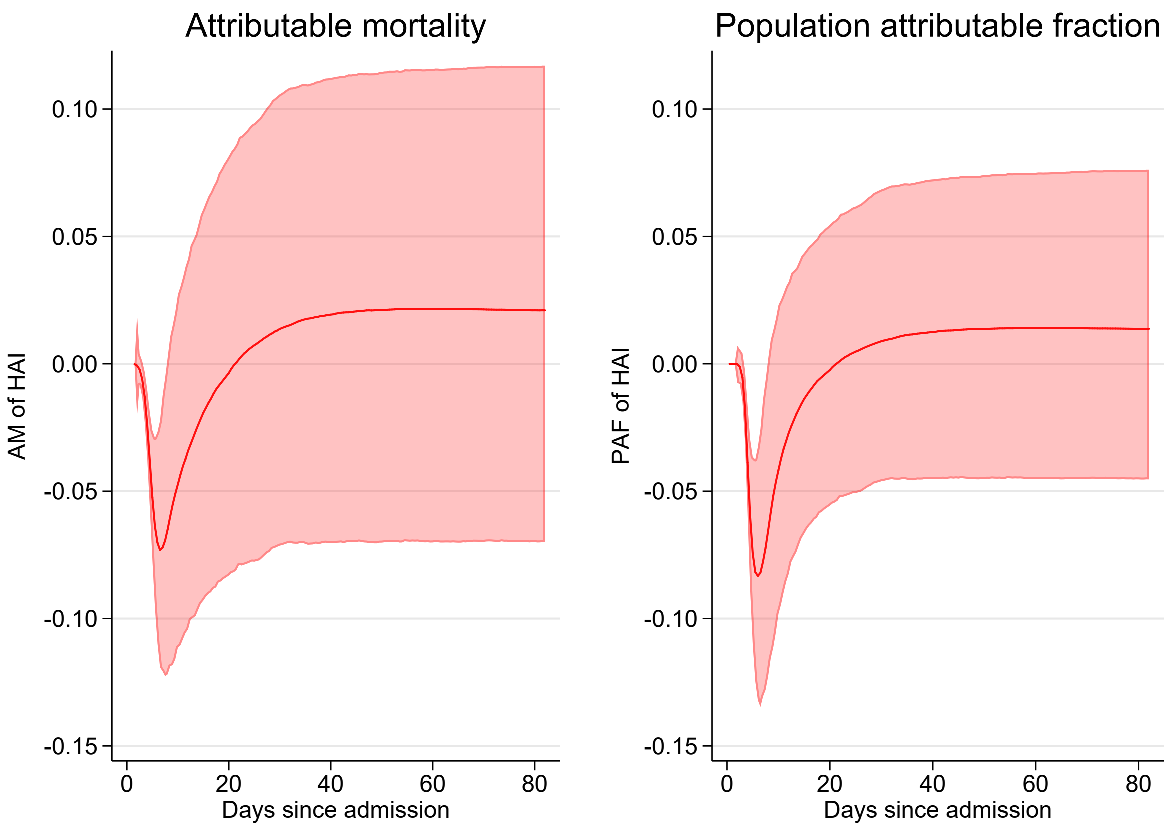

Supplementary Figure 5: Attributable mortality (AM) and population attributable fraction (PAF) of HAIs for the "AIC" model (solid line) with 95% confidence intervals (shaded region)

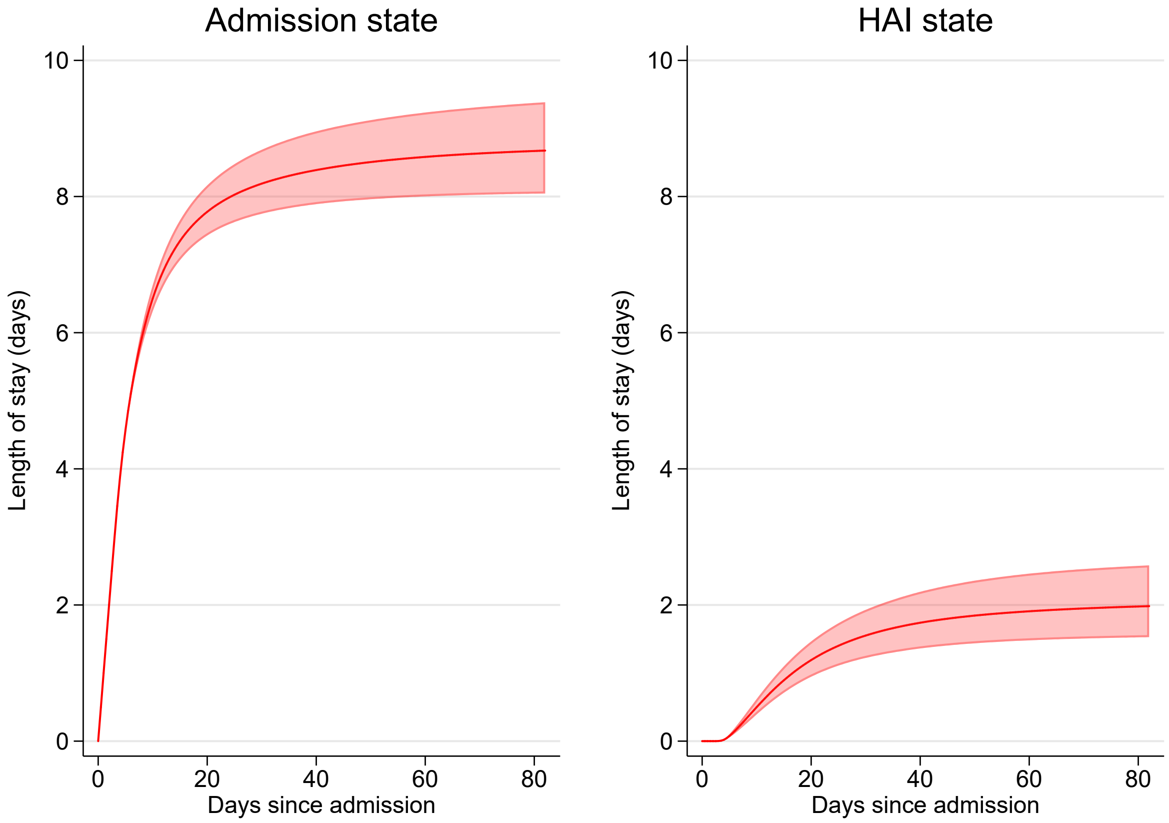

Supplementary Figure 6: Length of stay in hospital without (state 1, left panel) and with (state 2, right panel) a HAI starting from state 1 at time 0 for the "AIC" model (solid line) with 95% confidence intervals (shaded region)

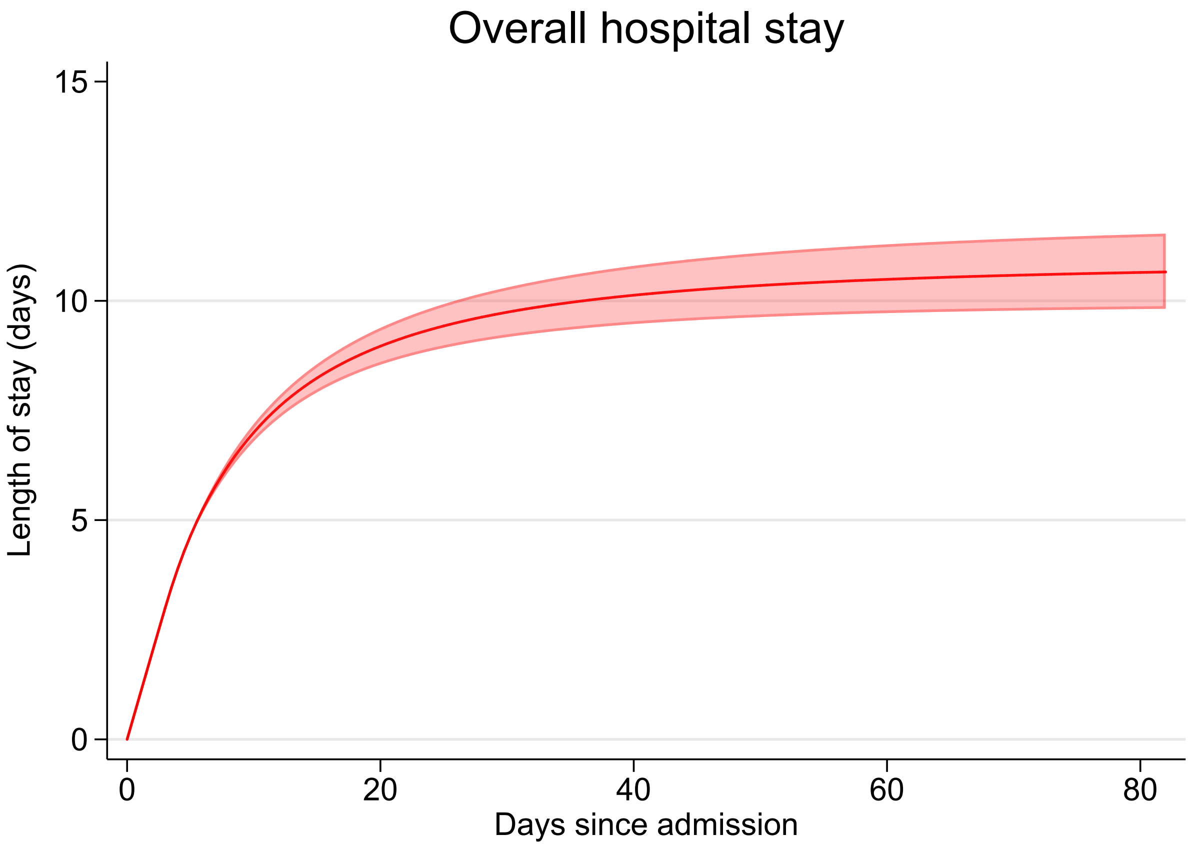

Supplementary Figure 7: Total length of stay in hospital from state 1 at time 0 for the "AIC" model (solid line) with 95% confidence intervals (shaded region)
